# Supplementary material for: Novel Phenotypical and Biochemical Findings in Mucolipidosis Type II
Source: Int J Mol Sci. 2025 Mar 7;26(6):2408. doi: 10.3390/ijms26062408 (PMC11941985; doi:10.3390/ijms26062408)
Supplement: Supplementary file 1 [file ijms-26-02408-s001.zip › ijms-3477053-supplementary.pdf]

Novel Phenotypical and Biochemical Findings in Mucolipidosis Type II

Eines Monteagudo-Vilavedra [1], Daniel Rodrigues [1,2], Giorgia Vella [1], Susana B. Bravo [3], Carmen Pena [4], Laura Lopez-Valverde [1,2], Cristobal Colon [1,2], Paula Sanchez-Pintos [1,2,5], Francisco J. Otero Espinar [6,7], Maria L. Couce [1,2,3,*] and J. Victor Alvarez [1,2,*]

Proteomic analysis by TripleTOF 6600 LC-MS/MS System

Protein preparation from Dried Blood Samples

Protein from **Dried Blood Samples** was extracted by incubating the paper in a 100 µl of 100 mM ammonium bicarbonate at room temperature for 1h. The sample was centrifuge 20 min at 13,000 xg, and the supernatant was transferred to a new tube. Then the protein was precipitated by MeOH/ChCl3 method, and the protein concentration was measure using a RC-DC kit (BioRad)

Protein digestion

In order to perform global protein identification and quantification, an equal amount of protein from each sample was loaded on a 10% SDS-PAGE gel. The run was stopped as soon as the front had penetrated 3 mm into the resolving gel (36,37). The protein bands were detected by Sypro Ruby fluorescent staining (Lonza, Switzerland), excised, and processed for in-gel, manual tryptic digestion as described elsewhere (39-40). Gel pieces were reduced with 10 mM dithiothreitol (Sigma-Aldrich, St. Louis, MO) in 50 mM ammonium bicarbonate (Sigma-Aldrich, St. Louis, MO) and alkylated with 55 mM iodoacetamide (Sigma-Aldrich, St. Louis, MO) in 50 mM ammonium bicarbonate. Then, gel pieces were rinsed with 50 mM ammonium bicarbonate in 50% methanol (HPLC grade, Scharlau, Barcelona, Spain), dehydrated by addition of acetonitrile (HPLC grade, Scharlau, Barcelona, Spain), and dried in a SpeedVac. Modified porcine trypsin (Promega, Madison, WI, USA) was added to the dry gel pieces at a final concentration of 20 ng/µl in 20mM ammonium bicarbonate, incubating them at 37 °C for 16 h. Peptides were extracted

thrice by 20 min incubation in 40 µL of 60% acetonitrile in 0.5% HCOOH. The resulting peptide extracts were pooled, concentrated in a SpeedVac, and stored at −20 °C.

Creation of the spectral library

In order to build the MS2 (MS/MS spectral libraries) spectral libraries, the peptide solutions were analyzed by a shotgun **data-dependent acquisition (DDA)** approach using micro-LC-MS/MS. To obtain a good representation of the peptides and proteins present in all samples 4 µL of each sample was separated into a micro-LC system Ekspert nLC425 (Eksigen, Dublin, CA, USA) using an Eksigent C18 150 × 0.30 mm, 3 mm particle size and 120 Å pore size (Eksigent, Sciex) at a flow rate of 5µL/min. Water and ACN, both containing 0.1% formic acid, was used as solvents A and B, respectively. The gradient run consisted of 5% to 95% B for 30 min, 5 min at 90% B and finally 5 min at 5% B for column equilibration, for a total run time of 40 min. As the peptides eluted, they were directly injected into a hybrid quadrupole-TOF mass spectrometer Triple TOF 6600 (Sciex, Redwood City, CA, USA) operated with a data-dependent acquisition system in positive ion mode. A Micro source (Sciex) was used for the interface between microLC and MS, with an application of 2600 V voltage. The acquisition mode consisted of a 250 ms survey (MS scan) MS1 scan from 400 to 1250 m/z followed by an (MSMS Scan) MS2 scan from 100 to 1500 m/z (25 ms acquisition time) of the top 65 precursor ions from the survey scan, for a total cycle time of 2.8 s. The fragmented precursors were then added to a dynamic exclusion list for 15 s; any singly charged ions were excluded from the (MS/MS analysis) MS2 analysis.

The peptide and protein identifications was performed using Protein Pilot software (version 5.0.1, Sciex) with a Data was searched using a Human specific Uniprot database, specifying iodoacetamide as Cys alkylation and metionin oxidation as fixed modification. The false discovery rate (FDR) was set to 1 for both peptides and proteins. The MS2 spectra (MS/MS spectra) of the identified peptides were then used to

generate the spectral library for SWATH peak extraction using the add-in for PeakView Software (version 2.2, Sciex) MS/MSALL with SWATH Acquisition MicroApp (version 2.0, Sciex). Peptides with a confidence score above 99% (as obtained from Protein Pilot database search) were included in the spectral library).

Relative quantification by SWATH acquisition

SWATH (Sequential Window Acquisition of all Theoretical Mass Spectra) – MS acquisition was performed on a TripleTOF® 6600 LC-MS/MS system (Sciex). 4 µL of Peptides from each individual samples run as triplicated were analyzed using a **data-independent acquisition (IDA)** method. In this case a LC-MS equipment and LC gradient described above for building the spectral library but instead using the SWATH-MS acquisition method. The method consisted of repeating a cycle that consisted of the acquisition of 100 TOF (MS/MS scans) MS2 scans (400 to 1500 m/z, high sensitivity mode, 50 ms acquisition time) of overlapping sequential precursor isolation windows of variable width (1 m/z overlap) covering the 400 to 1250 m/z mass range with a previous TOF MS1 scan (400 to 1500 m/z, 50 ms acquisition time) for each cycle. Total cycle time was 6.3 s. For each sample set, the width of the 100 variable windows was optimized according to the ion density found in the DDA runs using a SWATH variable window calculator worksheet from Sciex.

Data analysis

The targeted data extraction of the fragment ion chromatogram traces from the SWATH runs was performed by PeakView (version 2.2, Sciex) using the SWATH Acquisition MicroApp(version 2.0). This application processed the data using the spectral library created from the shotgun data. Up to ten peptides per protein and seven fragments per peptide were selected, based on signal intensity; any shared and modified peptides were excluded from the processing. Five minute windows and 30 ppm widths was used to extract the ion chromatograms; SWATH quantization was attempted for all proteins in the ion library

that was identified by ProteinPilot with an FDR below 1%. The retention times from the peptides that were selected for each protein was realigned in each run according to the iRT peptides corresponding a different identified proteins in each sample and eluted along the whole time axis. The extracted ion chromatograms were then generated for each selected fragment ion; the peak areas for the protein were obtained by summing the peak areas from 10 peptides (MS1 scan) and 7 corresponding fragment ions (MS2 scan) from each peptide. PeakView computed an FDR and a score for each assigned peptide according to the chromatographic and spectra components; only peptides with an FDR below 5% were used for protein quantization. Protein quantization was calculated by adding the peak areas of the corresponding peptides. The integrated peak areas (processed. mrkvw files from PeakView) were directly exported to the MarkerView software (Sciex) for relative quantitative analysis. The export will generate three files containing quantitative information about individual ions, the summed intensity of different ions for a particular peptide and the summed intensity of different peptides for a particular protein. MarkerView uses processing algorithms that accurately find chromatographic and spectral peaks direct from the raw SWATH data. Data alignment by MarkerView compensates for minor variations in both mass and retention time values, ensuring that identical compounds in different samples are accurately compared to one another. For the protein/peptide library, its set of differentially expressed proteins (FC>1.5 or FC< 0.6 and p-value <0.05) up regulated or down regulated proteins was selected

Functional and pathway analysis

Most significant pathways were represented using Reactome pathway diagrams. Protein interactions were evaluated using String (https://string-db.org/), considering a minimum required interaction score of PPI=0.9 (protein protein interaction) and a FDR <0.05. Venn diagrams were generated using http://www.interactivenn.net/ and box plots using GraphPad Prism 9. Statistical analyses were performed using Scaffold software.
